# MicroRNA-206 as a potential cholesterol-lowering drug is superior to statins in mice

**DOI:** 10.1016/j.jlr.2024.100576

**Published:** 2024-06-10

**Authors:** Chao Li, Jing Tian, Ningning Liu, David Song, Clifford J. Steer, Qinghua Han, Guisheng Song

**Affiliations:** 1 Department of Cardiology, The First Hospital of Shanxi Medical University, Taiyuan City, China; 2 The First College of Clinical Medicine, Shanxi Medical University, Taiyuan City, China; 3 Department of Medicine, University of Minnesota, Minneapolis, MN, USA

**Keywords:** hypercholesterolemia, microRNA, cholesterol-lowering medications, statins, MAFLD, adverse effects

## Abstract

Hypercholesterolemia is frequently intertwined with hepatosteatosis, hypertriglyceridemia, and hyperglycemia. This study is designed to assess the therapeutic efficacy of miR-206 in contrast to statins in the context of managing hypercholesterolemia in mice. We previously showed that miR-206 is a potent inhibitor of de novo lipogenesis (DNL), cholesterol synthesis, and gluconeogenesis in mice. Given that these processes occur within hepatocytes, we employed a mini-circle (MC) system to deliver miR-206 specifically to hepatocytes (designated as MC-miR-206). A single intravenous injection of MC-miR-206 maintained high levels of miR-206 in the liver for at least two weeks, thereby maintaining suppression of hepatic DNL, cholesterol synthesis, and gluconeogenesis. MC-miR-206 significantly reduced DNA damage, endoplasmic reticulum and oxidative stress, and hepatic toxicity. Therapeutically, both MC-miR-206 and statins significantly reduced total serum cholesterol and triglycerides as well as LDL cholesterol and VLDL cholesterol in mice maintained on the normal chow and high-fat high-cholesterol diet. MC-miR-206 reduced liver weight, hepatic triglycerides and cholesterol, and blood glucose, while statins slightly increased hepatic cholesterol and blood glucose and failed to affect levels of liver weight and hepatic triglycerides. Mechanistically, miR-206 alleviated hypercholesterolemia by inhibiting hepatic cholesterol synthesis, while statins increased HMGCR activity, hepatic cholesterol synthesis, and fecal-neutral steroid excretion. MiR-206 facilitates the regression of hypercholesterolemia, hypertriglyceridemia, hyperglycemia, and hepatosteatosis. MiR-206 outperforms statins by reducing hyperglycemia, hepatic cholesterol levels, and hepatic toxicity.

Hyperlipidemia is a major risk factor for cardiovascular disease (CVD) and fatal strokes. Hyperlipidemia is classified, according to the type of lipid elevated, by hypercholesterolemia, hypertriglyceridemia, or both. ∼ 50% of adults over the age of 20 years have hypercholesterolemia, and 31% of them have hypertriglyceridemia. (1, 2) This lipid profile significantly increases the risk of cardiovascular events and strokes, particularly when coupled with related comorbidities. For the treatment of hyperlipidemia, lifestyle change alone is often unsuccessful. In addition, patients who require lipid-lowering agents are often noncompliant. It has been reported that ∼ 50% of patients taking lipid-lowering drugs discontinue their medication after 1 year, and 75% stop after 2 years. (3, 4) Statins, as a predominant therapeutic approach for hypercholesterolemia, can only mitigate the risk of coronary artery disease by 20%–40%. (5) For patients with heart failure or other end-stage diseases, the benefits of statins are exceedingly limited. (6) Many types of adverse effects of statins have been reported including liver damage, hyperglycemia, weight gain, headache, difficulty sleeping, muscle pain, and dizziness. (7) Novel and effective therapeutic strategies for managing hyperlipidemia are not just essential but also urgently needed.

The discovery of a class of small non-coding RNAs, termed microRNAs (miRNAs), has stimulated a new field of hyperlipidemia therapy. (8) MiRNAs precisely fine-tune gene expression by binding to the 3′-untranslated regions (3′UTR) of mRNAs. (8) MiRNAs can target multiple genes. (8) During the evolution, a stable interactome between miRNAs and their targets is formed to maintain physiological homeostasis. (9, 10, 11, 12) Notably, hypercholesterolemia often occurs with hypertriglyceridemia, hepatosteatosis, and hyperglycemia, (1, 2) indicating the necessity to develop a therapeutic drug against these interconnected metabolic disorders. The exquisite characteristics of miRNAs as described above make them an ideal regulator to precisely regulate lipid and lipoprotein homeostasis by simultaneously modulating several pathways. Given the relative ease of manufacturing inhibitors and mimics of miRNAs, they now represent novel therapeutic agents for a growing list of human diseases. (13, 14, 15, 16). Indeed, the first miRNA-based therapeutic agent for HCV infection has entered into a phase III clinical trial. (17) All these findings urged us to identify miRNA-based medications against highly associated hypertriglyceridemia, hepatosteatosis, and hypercholesterolemia. In our earlier investigations, we demonstrated that miR-206 has the capacity to improve hepatic insulin resistance, promote glycolysis, and inhibit gluconeogenesis by targeting *Ptpn1* (protein tyrosine phosphatase non-receptor type 1). (18) Notably, miR-206 emerges as a strong inhibitor of de novo lipogenesis (DNL) and cholesterol synthesis by targeting *Hmgcr* (3-hydroxy-3-methylglutaryl-CoA reductase) and the PTPN1-Srebp1c axis. (18, 19) Furthermore, miR-206 functions as a potent tumor suppressor by directly targeting *Ccnd1* (cyclin D), *Notch3*, and *Kras* in hepatocytes and *Klf4* (Krüppel-like factor 4) in Kupffer cells (KCs). (20, 21, 22, 23) All of these findings indicate the robust therapeutic potential of miR-206. Statins as the first-line medication against hypercholesterolemia are designed based on HMGCR. Given the adverse effects of statins and their ineffectiveness on hepatosteatosis and hyperglycemia that often occurs with hypercholesterolemia, in this study, we used mini-circles (MC) as a vehicle to deliver miR-206 to the liver and compared the effects of statins and miR-206 on hypercholesterolemia, hepatosteatosis, hypertriglyceridemia, and hyperglycemia.

## Materials and methods

### Statement on institutional approval for mice experimentation

Eight-week-old wild-type male C57BL/6J mice (Jackson Laboratory) were used for experiments. Mice were housed in a barrier facility on 12 h:12 h light cycle with free access to water and a normal chow (Open Source D12450B: 10% Kcal fat) or an HFHC diet (Open Source D12108C: 40% Kcal fat and 1.25% cholesterol). Animal care, plasmid injection, and surgical procedures were conducted in compliance with an approved IACUC protocol by the University of Minnesota and Shanxi Medical University.

### Preparation of mini-circle expression vector for miR-206

We generated an in vivo expression vector of miR-206 by cloning human miR-206 precursor into mini-circle vectors (MC) purchased from System Biosciences (Cat. MN511A-1). A transthyretin gene (TTR) promoter was inserted into the upstream of miR-206 precursor to ensure liver-specific expression of miR-206 (MC-miR-206). To rule out non-specific effects of the plasmid, we generated an MC-based scramble expression vector (MC-SCR). The parental MC-miR-206 vector was transformed into a special host *E. coli* bacterial strain ZYCY10P3S2T (System Biosciences, Cat: MN900A-1). Mini-circles were generated based on the manufacturer’s instructions.

### Injection of MC-miR-206

Eight-week-old wild-type male C57BL/6J mice (Jackson Laboratory, n = 6) were maintained on the HFHC diet (Open Source D12108C: 40% Kcal fat and 1.25% cholesterol) for 8 weeks. At 16 weeks of age, mice received a dose of 2 μg/g MC-miR-206 or MC-SCR (control) weekly for 8 weeks via tail vein injection. At that time point, mice were fasted for 8 h. Livers and blood were harvested for further analysis.

### Treatment of rosuvastatin, lovastatin and MC-miR-206

The HFHC diet was supplemented with 0.01% rosuvastatin or 0.2% lovastatin. These doses were chosen based on previous publications for use in mice without obvious toxic side effects. (24, 25, 26, 27) Eight-week-old wild-type male C57BL/6J mice were maintained on the HFHC diet for 8 weeks. At 16 weeks of age, mice were randomly allocated into four groups (n = 6). Group I was continued to be kept on the HFHC and injected with MC-SCR (n = 6) weekly for 8 weeks; Groups II (n = 6) and III (n = 6) were maintained the HFHC supplemented with 0.01% rosuvastatin or 0.2% lovastatin for 8 weeks; and Group IV (n = 6) was kept on the HFHC and injected with MC-miR-206 weekly for 8 weeks.

The normal chow was supplemented with 0.01% rosuvastatin or 0.2% lovastatin. Eight-week-old wild-type male C57BL/6J mice were randomly allocated into four groups (n = 6). Group I was injected with MC-SCR (n = 6) weekly for eight weeks; Groups II (n = 6) and III (n = 6) were maintained the normal chow supplemented with 0.01% rosuvastatin or 0.2% lovastatin for eight weeks; and Group IV (n = 6) was injected with MC-miR-206 weekly for eight weeks.

### Cholesterol synthesis assay

Eight-week-old wild-type male C57BL/6J mice kept on the HFHC diet or normal chow were treated with statins or MC-miR-206 for 2 weeks. After 2 weeks of statin or MC-miR-206 treatment, 2% ^13^C-acetate was added to the drinking water ad libitum for 72 h. Bloodspots were taken at time points 0, 24, 31, 48, 55, and 72 h. Mice were terminated under anesthesia by cardiac puncture, and consecutively, livers were excised. Cholesterol extraction was performed as previously described. (28) Fractional cholesterol synthesis was determined by mass isotopomer distribution analysis using ^13^C-acetate (Sigma) as a labeled precursor based on the method described previously. (29) The incorporation of ^13^C into newly synthesized cholesterol molecules was quantified via gas chromatography-mass spectrometry (GC/MS). Cholesterol synthesis was calculated as detailed previously. (28, 29, 30).

To determine cholesterol synthesis in the liver, mice were injected with 99% deuterium oxide (23.3 mg/g; ip) after 2 weeks of statins or MC-miR-206 treatment. Mice were terminated by cardiac puncture 60 min after injection. Cholesterol extraction from the liver tissues was performed as described above. Liver-specific cholesterol synthesis in deuterium water-injected mice was analyzed via the method previously described. (28, 31).

### LDL Uptake Assay Kit

Primary hepatocytes were isolated from mice by perfusion as described previously. (20, 22) The rates of hepatic LDL uptake were performed using the LDL Uptake Assay Kit (Cell-Based) according to the manufacturer’s protocol (BioVision: Catalog^#^ K436-100).

### Statistical analysis

Data were analyzed using Prism Software® (GraphPad). Data represent mean ± SD. The sample size was determined by power analysis to provide sufficient statistical power to detect differences. Two-tailed student’s *t* test was used to compare two datasets and one-way ANOVA (analysis of variance) analysis was performed to compare three or more datasets. *P* < 0.05 was considered statistically significant.

## Results

### MC-mediated sustained and elevated miR-206 expression in hepatocytes

**Fig. 1:**
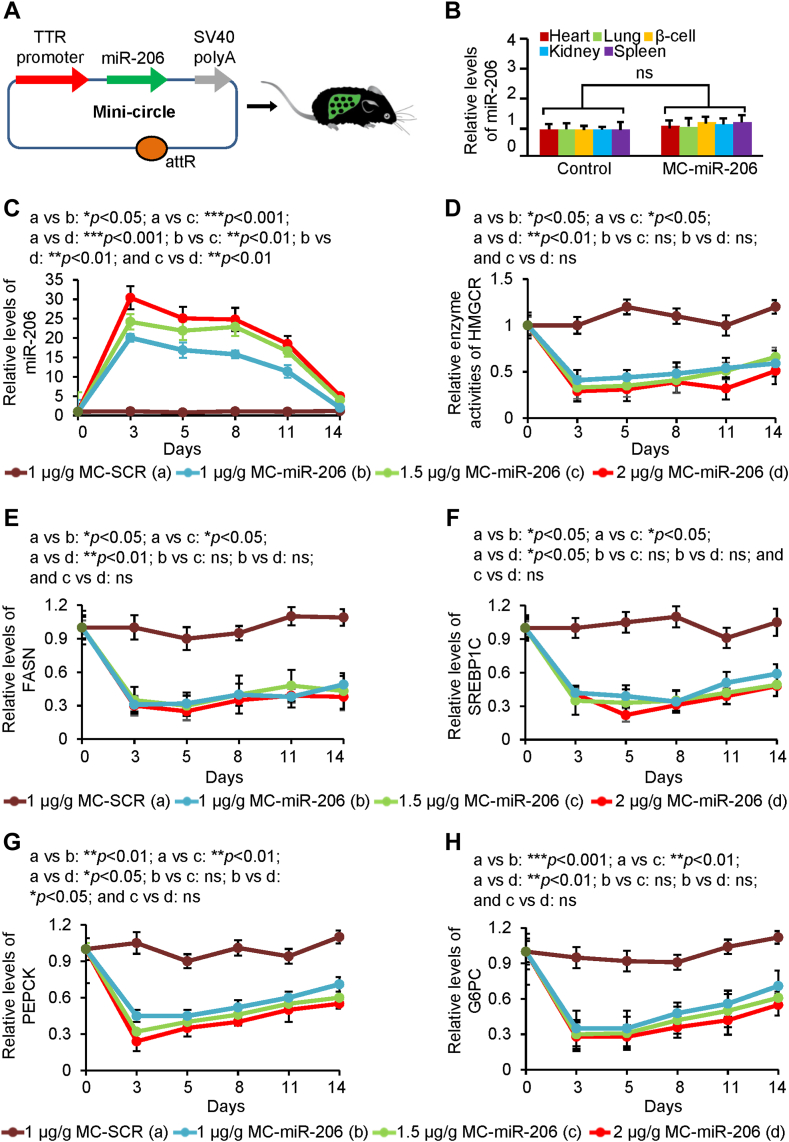
Dosage effect of MC-miR-206 on its targets. A: Diagram of miR-206 mini-circle expression vector (MC-miR-206). *TTR* promoter was used to ensure hepatocyte-specific expression of miR-206. B: Levels of miR-206 in heart, lung, β-cell, kidney and spleen of mice injected with MC-SCR (n = 6) or MC-miR-206 (n = 6) at a dose of 2 μg/g body weight via tail vein. Eight-week-old male C57BL/6J mice were maintained on the normal chow. C–H: Levels of miR-206, HMGCR enzyme activity and protein level of FASN, SREP1C, PEPCK or G6PC in livers of mice injected with MC-miR-206 at a dose of 1 μg/g (n = 6), 1.5 μg/g (n = 6), or 2 μg/g (n = 6) (body weight). Eight-week-old male C57BL/6J mice were maintained on the normal chow were injected with MC-miR-206 via tail-vein. After a 2-weeks post-injection period, mice were euthanized, and their livers were collected for the assessment of HMGCR enzyme activity and protein levels of SREP1C, FASN, G6PC, and PEPCK via ELISA. Data represent mean ± SD. ns: no significance (B: two-tailed student’s *t* test and C–H: one-way ANOVA test).

To efficiently deliver miR-206 to the liver, we generated a miR-206 in vivo expression vector using a mini-circle (MC) episomal DNA vector. (32) MCs are episomal DNA vectors that are produced as circular expression cassettes devoid of any bacterial plasmid DNA backbone. (33) Their smaller molecular size enables more efficient delivery and offers sustained expression over a period of weeks as compared to standard plasmid vectors that only work for a few days after injection into mice. We cloned the human miR-206 precursor into the MC parental plasmid. *TTR* (transthyretin) promoter was used to ensure liver-specific expression of miR-206. (34) This construct was referred to as MC-miR-206 (Fig. 1A). MC-miR-206 was dissolved in saline at a concentration of 1 μg/μl. We next injected wild-type C57BL/6J mice maintained on the normal chow with MC-miR-206 or MC-scramble (MC-SCR) at a concentration of 1 μg/g, 1.5 μg/g and 2 μg/g body weight in a total volume of 100 μl saline via tail-vein. Our mechanistic studies have established that miR-206 is a strong inhibitor of cholesterol synthesis, gluconeogenesis, and DNL by inhibiting genes encoding HMGCR, G6PC (glucose-6-phosphatase catalytic-subunit), PEPCK (phosphoenolpyruvate carboxykinase), FASN (fatty acid synthase) and Srebp1. (18, 19) HMGCR is the rate-limiting enzyme of cholesterol synthesis; G6PC and PEPCK are the rate-limiting enzymes of gluconeogenesis; and FASN is the rate-limiting enzyme of DNL. (18, 19) At 3, 5, 8, 11, and 14 days after injection, we measured levels of miR-206, HMGCR enzyme activity, and protein levels of FASN, G6PC, PEPCK, and SREBP1C in hepatocytes of mice. Injection of MC-miR-206 into mice led to high levels of miR-206 in the liver, but no significant change in other tissues (Fig. 1B). A single injection of MC-miR-206 maintained high levels of miR-206 in the liver for two weeks (Fig. 1C). Consistent with increased miR-206, HMGCR activity and protein levels of FASN and SREBP1C were significantly reduced in MC-miR-206-injected mice (Fig. 1D–F). We have previously established that miR-206 inhibits gluconeogenesis by inhibiting G6PC and PEPCK, two rate-limiting enzymes of gluconeogenesis. (18) Protein levels of PEPCK and G6PC were significantly reduced in MC-miR-206-injected mice (Fig. 1G, H). In sum, a single injection of MC-miR-206 is able to maintain low levels of HMGCR, FASN and SREPB1C, G6PC, and PEPCK in the liver for 2 weeks. MC-miR-206 demonstrates the absence of off-target miR-206 expression in other organs.

### MC-miR-206 treatment alleviated DNA damage, endoplasmic reticulum and oxidative stress, and hepatic toxicity

**Fig. 2:**
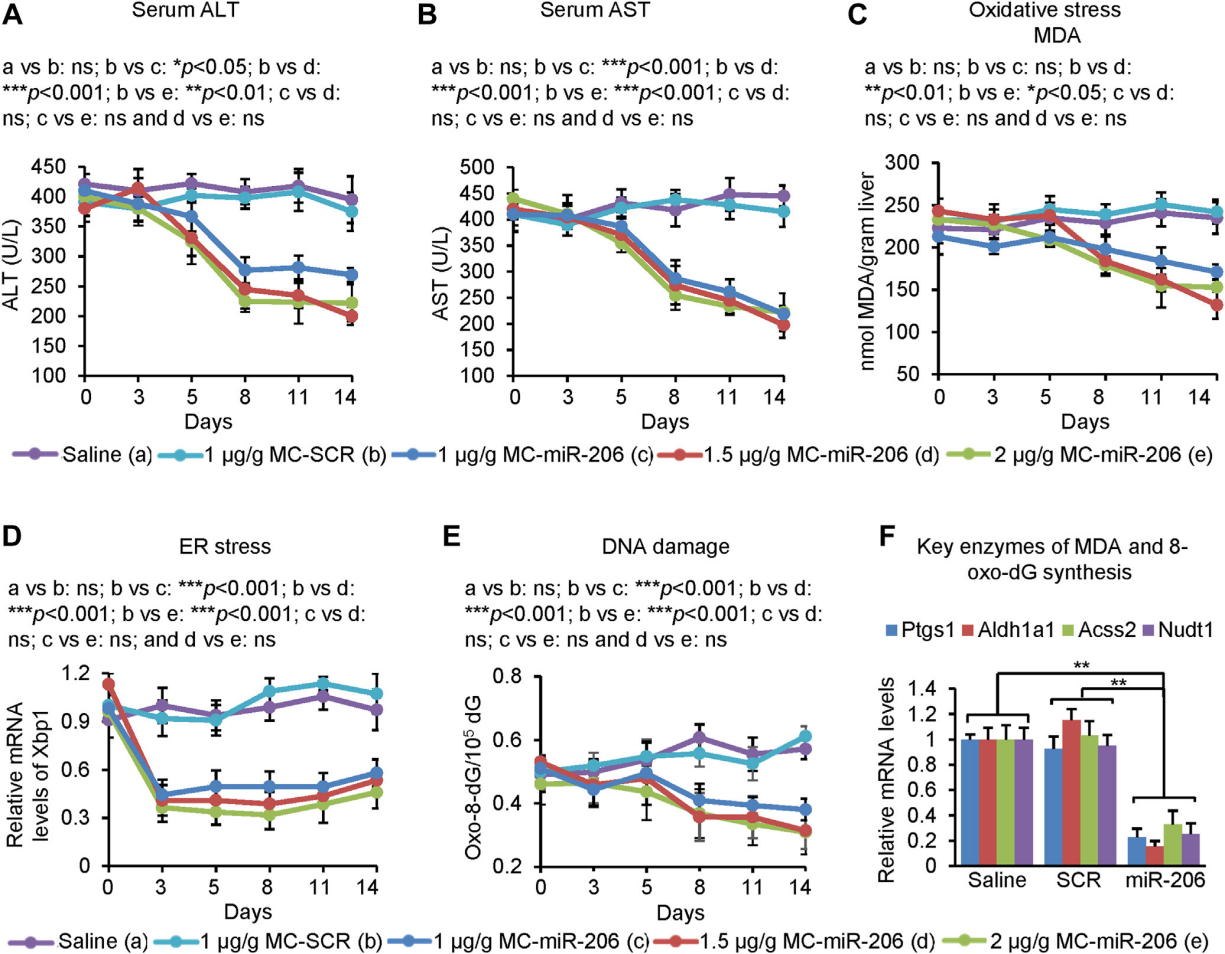
MC-miR-206 alleviated hepatic toxicity, ER and oxidative stress and DNA damage. Eight-week-old male C57BL/6J mice were maintained on the HFHC diet for eight weeks and then were injected with saline (n = 6), MC-SCR (n = 6) or MC-miR-206 at a dose of 1 μg/g (n = 6), 1.5 μg/g (n = 6), or 2 μg/g (n = 6) via tail-vein. After an eight-week post-injection period, mice were euthanized. Their livers were collected for analyzing gene expression and levels of hepatic MDA and oxo-8-dG. A, B: Levels of serum ALT and AST. C–E: Levels of hepatic MDA, *Xbp1* mRNA, and oxo-8-dG. F: mRNA levels of genes encoding PTGS1, ALDH1A1, ACSS2 and NUDT1 in livers. Data represent mean ± SD. ∗∗∗*P* < 0.001 and ns: no significance (A–F: one-way ANOVA test).

Drug-induced liver injury represents a significant clinical concern. Statins use has been associated with elevations in serum ALT levels in approximately 3% of persons who take the drugs. (35, 36) We, therefore, evaluated the effect of MC-miR-206 on DNA damage, endoplasmic reticulum (ER) oxidative stress, and hepatic toxicity. Eight-week-old C57BL/6J mice were kept on the HFHC diet for eight weeks. At 16 weeks of age, mice were treated with saline, MC-SCR, and various doses of MC-miR-206. Serum chemistry revealed that MC-miR-206 treatment significantly reduced hepatic injury, which was reflected by a significant reduction in ALT and AST levels in miR-206 treated mice (Fig. 2A, B). Transcription of gene encoding XBP1 (X-box binding protein 1) is induced in response to ER stress. (37) Malondialdehyde (MDA) level is commonly known as a marker of oxidative stress in subjects with familial hypercholesterolemia. (38) In addition to lowering ALT and AST levels, miR-206 markedly decreased hepatic MDA levels and downregulated expression of *Xbp1* (Fig. 2C, D). 8-Oxo-dG is one of the major products of DNA oxidation, which induces intramolecular DNA damage. (39) MC-miR-206 treatment significantly reduced levels of 8-Oxo-dG (Fig. 2E). We next analyzed the expression of genes controlling synthesis of 8-Oxo-dG and MDA. Consistent with reduced MDA, MC-miR-206 led to an five-fold decrease in the expression of genes encoding PTGS1 (prostaglandin-endoperoxide synthase 1), ALDH1A1 (aldehyde dehydrogenase 1 family member A1) and ACSS2 (acetate-dependent acetyl-CoA synthetase 2) (Fig. 2F), three critical enzymes of MDA synthesis. (40) MC-miR-206 also significantly reduced the expression of *Nudt1* (nudix hydrolase 1) (Fig. 2F), a gene controlling 8-Oxo-dG production. (41) In sum, MC-miR-206 reduced hepatic toxicity, ER and oxidative stress, and DNA damage.

### MC-miR-206 induced a substantial reduction in hepatic and serum triglycerides and cholesterol, along with lowered blood glucose and reduced levels of LDL/VLDL cholesterol

**Fig. 3:**
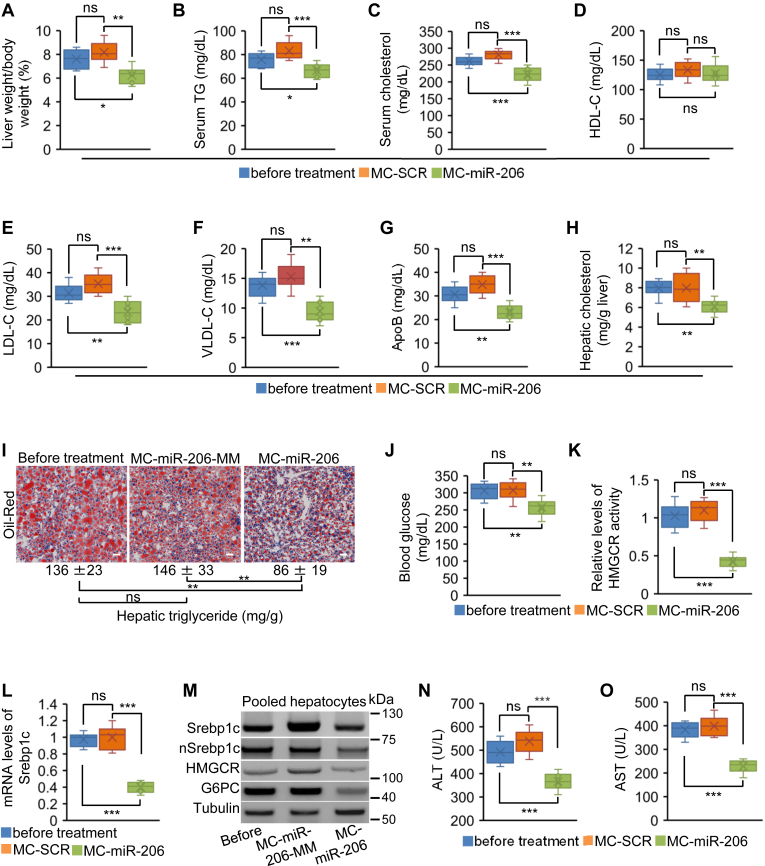
MC-miR-206 drove a regression of hypercholesterolemia, hyperglycemia, hypertriglyceridemia and hepatosteatosis. Eight-week-old C57BL/6J male mice were kept on the HFHC diet for eight weeks. At 16 weeks of age, six mice were sacrificed to establish pathological parameters before miR-206 treatment. The remaining mice were randomly allocated into two groups and injected with either MC-miR-206 (n = 6) or MC-SCR (control, n = 6) weekly for eight weeks. Eight weeks post injection, livers and blood were collected for the following analysis. A: Ratios of the liver weight to body weight; (B, C) Levels of total serum triglycerides (TG) and cholesterol; (D–F) Levels of HDL cholesterol (HDL-C), VLDL cholesterol (VLDL-C) and LDL cholesterol (LDL-C); (G) Levels of ApoB; (H, I) Levels of hepatic TG and cholesterol; (J) Levels of blood glucose; (K–M) Hepatic enzyme activity of HMGCR and protein levels of HMGCR, SREPB1C, nuclear SREBP1C and G6PC in three groups of mice; and (N, O) Levels of ALT and AST in three groups of mice. Scale bars in (I): 50 μm. Data represent mean ± SD. ∗*P* < 0.05, ∗∗*P* < 0.01, ∗∗∗*P* < 0.001 and ns: no significance (Fig. 3: one-way ANOVA test).

We next determined if MC-miR-206 could drive a regression in hepatic and serum triglycerides and cholesterol. Eight-week-old wild-type mice (C57BL/6J) were maintained on the HFHC diet for eight weeks. Six mice were then sacrificed to establish levels of hepatic triglycerides and cholesterol, circulating triglycerides and cholesterol, and blood glucose before MC-miR-206 injection. The remaining mice were randomly allocated into two groups of mice treated with MC-miR-206 or MC-SCR (control) weekly for eight weeks (a dose of 2 μg/g body weight in a total volume of 100 μl 0.9% NaCl) by tail vein injection. MC-miR-206 treatment led to a significant regression in the ratios of liver weight to body weight (Fig. 3A). MiR-206 treatment resulted in a regression in levels of total serum cholesterol and triglycerides (TG) (Fig. 3B, C). We next quantified levels of circulating HDL cholesterol (HDL-C), VLDL cholesterol (VLDL-C), and LDL cholesterol (LDL-C). No significant change in HDL-C was observed (Fig. 3D), while levels of both VLDL-C and LDL-C were significantly regressed in miR-206-treated mice (Fig. 3E, F). Consistently, serum levels of ApoB, a structural protein of LDL and VLDL, were lower than that in mice before miR-206-treatment (Fig. 3G). Hepatocytes are the major site for cholesterol and DNL. Considering the reduced serum TG and cholesterol in miR-206-treated mice, we further analyzed levels of hepatic TG and cholesterol in the livers of mice before and after treatment of miR-206. As expected, liver-specific expression of miR-206 promoted a significant regression of hepatic cholesterol and hepatosteatosis (Fig. 3H, I). Liver-specific expression of miR-206 also promoted a regression in hyperglycemia (Fig. 3J). Mechanistically, hepatocyte-specific expression of miR-206 led to significant regression of HMGCR activity; mRNA levels of *HMGCR*, *SREBP1C*, and *G6PC*; and protein levels of HMGCR, SREBP1C, nuclear SREBP1C (Nsrebp1c), and G6PC (Fig. 3K–M). MC-miR-206 also led to a significant regression in ALT and AST levels (Fig. 3N, O), indicating its ability to reduce hepatic toxicity. Together, MC-miR-206 contributed to a notable regression in hypercholesterolemia, hypertriglyceridemia, hyperglycemia, and hepatosteatosis while also providing significant relief from hepatic toxicity.

### Statins paradoxically increased but miR-206 significantly decreased cholesterol synthesis in hepatocytes

**Fig. 4:**
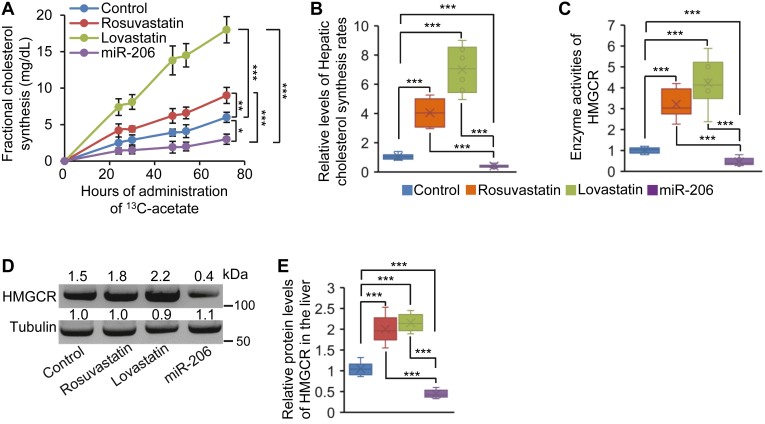
miR-206 inhibited but statins promoted hepatic cholesterol synthesis. A: Total body cholesterol synthesis measured by the incorporation of ^13^C-acetate into the cholesterol molecule over a period of 72 h in mice treated with MC-SCR (control, n = 6), rosuvastatin (n = 6), lovastatin (n = 6) or MC-miR-206 (n = 6). Eight-week-old wild-type male C57BL/6J mice kept on the HFHC diet were treated with statins or MC-miR-206 for two weeks. After two weeks of statin or MC-miR-206 treatment, 2% ^13^C-acetate was added to the drinking water for 72 h. Blood spots were taken at time points 0, 24, 31, 48, 55, and 72 h. B: Relative levels of hepatic cholesterol synthesis rates in four groups of mice, as revealed by deuterium oxide assay. Eight-week-old wild-type male C57BL/6J mice kept on the HFHC diet were treated with statins or MC-miR-206 for two weeks. Mice were then injected with 99% deuterium oxide. 60 min after injection, mice were terminated by cardiac puncture, livers were excised, and cholesterol extraction from the livers were performed. C: Enzyme activities of HMGCR in four groups of mice. D: Protein levels of HMGCR in pooled hepatocytes isolated from four groups of mice, as revealed by Western blot. E: Protein levels of HMGCR in hepatocytes isolated from four groups of mice as revealed by ELISA. Data represent mean ± SD. ∗*P* < 0.05, ∗∗*P* < 0.01 and ∗∗∗*P* < 0.001 (A–E: one-way ANOVA test).

We next compared the effects of statins and miR-206 on HMGCR activity and cholesterol synthesis. To evaluate cholesterol synthesis in vivo in response to statins and miR-206, eight-week-old wild-type male C57BL/6J mice kept on the HFHC diet were treated with statins or MC-miR-206 for 2 weeks. After that, ^13^C-acetate was added to the drinking water for 72 h. In contrast with the expectation of statins to inhibit hepatic cholesterol synthesis, treatment of rosuvastatin and lovastatin resulted in a significant increase in the rate of cholesterol synthesis (Fig. 4A). In contrast, miR-206 dramatically inhibited hepatic cholesterol synthesis (Fig. 4A). Some limitations of the use of ^13^C-acetate to measure cholesterol synthesis have been observed such as differences in cell entry of acetate and its conversion into acetyl-CoA among different types of cells and organs. (42) Deuterated water (D_2_O) can equilibrate with body water within minutes and therefore does not have this potential drawback. We next used D_2_O to determine hepatic cholesterol synthesis. D_2_O experiment confirmed that hepatic cholesterol synthesis was increased in statins-treated mice but reduced in miR-206-treated mice (Fig. 4B). Mechanistically, both enzyme activities and protein levels of HMGCR were significantly increased in statins-treated mice but significantly decreased in miR-206-treated mice (Fig. 4C–E). Given the inhibitory effect of the HFHC diet on cholesterol synthesis, we further analyzed the effects of miR-206 and statins on hepatic cholesterol synthesis in mice maintained on the normal chow. Consistent with the observation in mice maintained on the HFHC diet, miR-206 also significantly reduced hepatic cholesterol synthesis and levels of HMGCR protein and activity in the livers of mice kept on the normal chow (supplemental Fig. S1A–D). In sum, miR-206 inhibits cholesterol synthesis by reducing mRNA and protein levels of *Hmgcr*, while statins promote hepatic cholesterol synthesis by increasing the enzyme activity of HMGCR.

### MiR-206 is superior to statins in lowering hypercholesterolemia

**Fig. 5:**
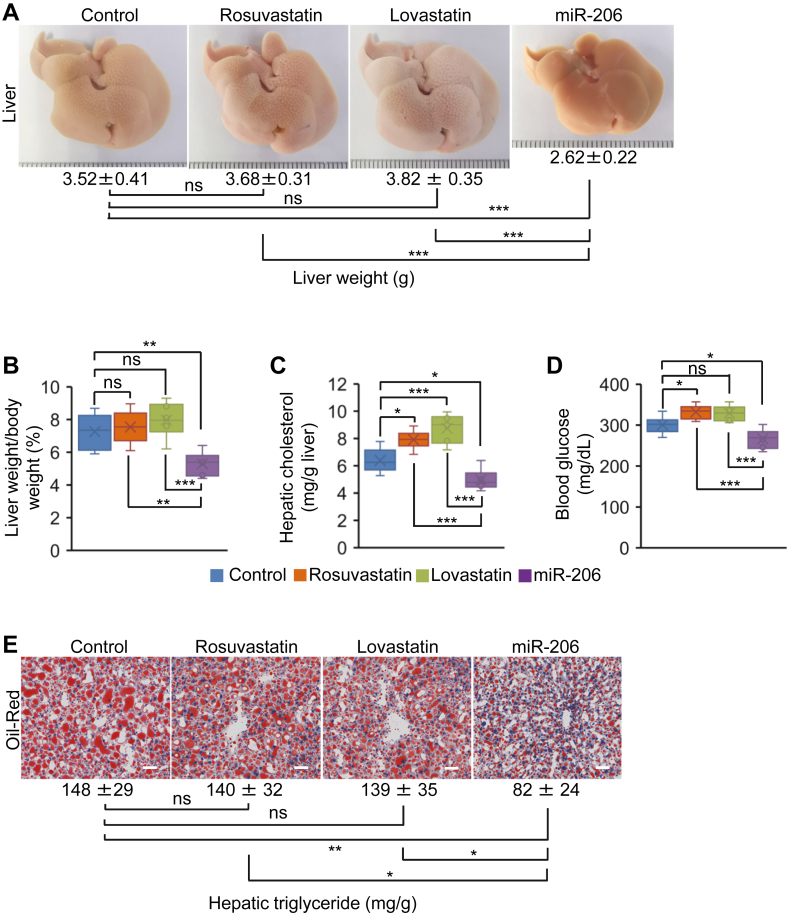
Statins increased hepatic cholesterol and blood glucose and exhibited no effect on hepatosteatosis, while miR-206 significantly reduced hepatic TG and cholesterol and blood glucose. Eight-week-old wild-type male C57BL/6J mice were kept on the HFHC diet for eight weeks. At 16 weeks of age, mice were injected with MC-SCR (control, n = 6), rosuvastatin (n = 6), lovastatin (n = 6), and MC-miR-206 (n = 6) for eight weeks. Livers and blood were collected. A, B: Representative photos of livers and the ratios of liver weight to body weight in four groups of mice. C–E: Levels of hepatic TG and cholesterol and blood glucose in four groups of mice. Scale bars in (E): 50 μm. Data represent mean ± SD. ∗*P* < 0.05, ∗∗*P* < 0.01, ∗∗∗*P* < 0.001 and ns: no significance (A–E: one-way AVOVA test).

**Fig. 6:**
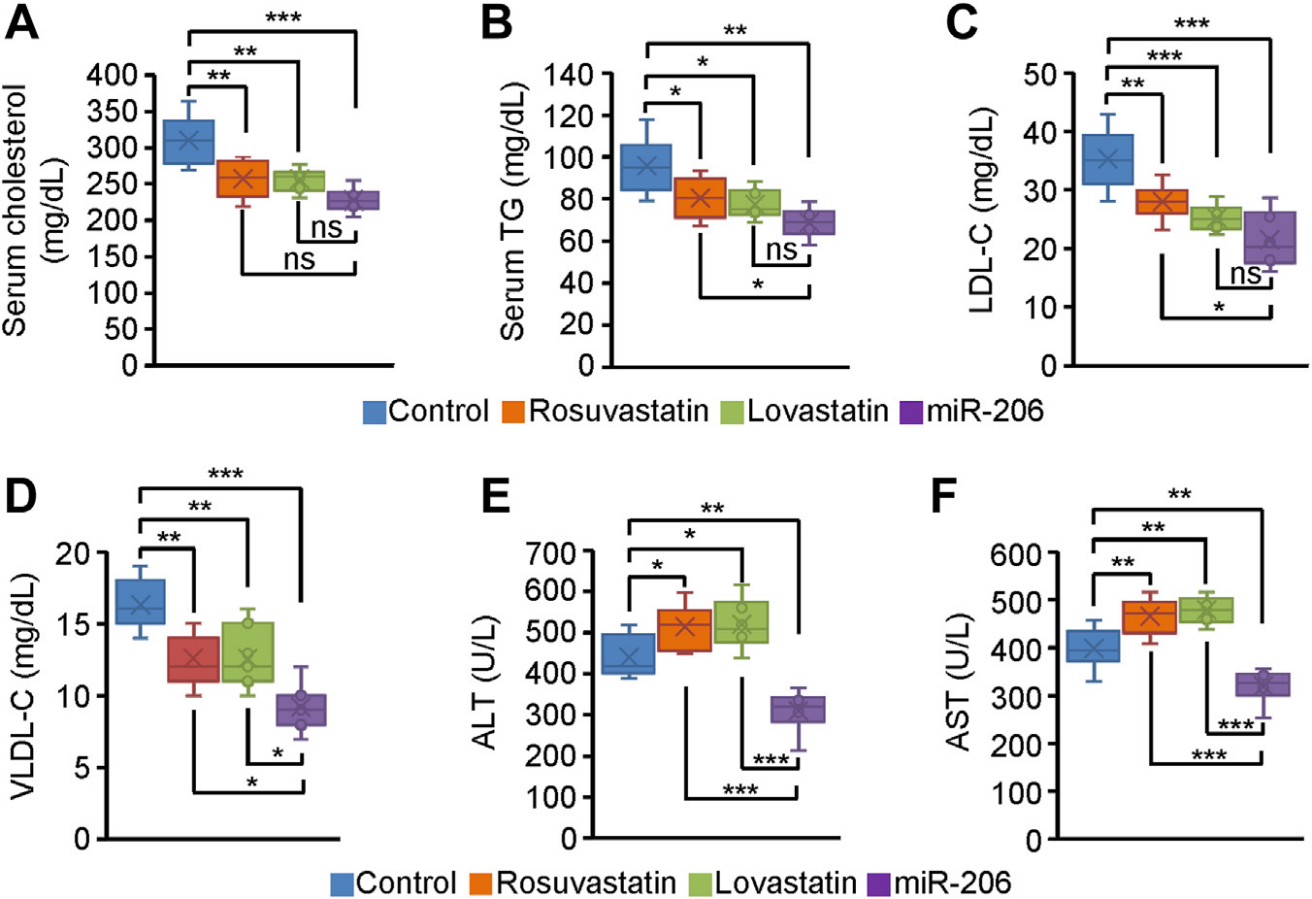
Both statins and miR-206 reduced serum TG and cholesterol as well as levels of VLDL-C and LDL-C. Eight-week-old wild-type male C57BL/6J mice were kept on the HFHC diet for eight weeks. At 16 weeks of age, mice were treated with MC-SCR (control, n = 6), rosuvastatin (n = 6), lovastatin (n = 6), and MC-miR-206 (n = 6) for eight weeks. A, B: Levels of total serum cholesterol and TG in four groups of mice treated with MC-SCR, rosuvastatin, lovastatin, and MC-miR-206. C, D: Levels of LDL-C and VLDL-C in four groups of mice. E, F: Levels of ALT and AST in four groups of mice. Data represent mean ± SD. ∗*P* < 0.05, ∗∗*P* < 0.01, ∗∗∗*P* < 0.001, and ns: no significance (A–F: one-way AVOVA test).

We next compared the therapeutic effects of miR-206 and statins on hypercholesterolemia. Specifically, four groups of mice kept on the HFHC diet were treated with MC-SCR, rosuvastatin, lovastatin, or MC-miR-206 for eight weeks. No significant change in the ratios of liver weight to body weight was observed in rosuvastatin and lovastatin-treated mice, while miR-206 significantly reduced the ratios of liver weight to body weight (Fig. 5A, B). Statins treatment significantly increased levels of hepatic cholesterol and blood glucose, in contrast to reduced hepatic cholesterol and blood glucose in miR-206-treated mice (Fig. 5C, D). MC-miR-206 treatment significantly reduced hepatic triglyceride content, but no significant change in hepatic TG was observed in statins-treated mice (Fig. 5E). Rosuvastatin was reported to reduce VLDL production in ApoE Leiden mice. (43) Both statins and miR-206 significantly reduced total serum cholesterol and TG, LDL-C and VLDL-C (Fig. 6A–D). Compared to increased ALT and AST levels in statins-treated mice, their levels were significantly reduced in miR-206-treated mice (Fig. 6E, F). In sum, both statins and miR-206 are able to reduce circulating triglyceride and cholesterol. As a potential therapeutic drug for hypercholesterolemia, miR-206 stands out for its clear advantages over statins. It actively reduces hepatic triglycerides and cholesterol, blood glucose, and hepatic toxicity. In contrast, the use of statins yields no benefits in addressing hepatosteatosis, and instead significantly contributes to heightened levels of hepatic cholesterol, and blood glucose, along with elevated AST and ALT levels.

Taking into account the impact of the HFHC diet on cholesterol synthesis, our subsequent investigation sought to compare how miR-206 and statins influence cholesterol homeostasis in mice maintained on the normal chow. No significant change in the ratios of liver weight/body weight was observed in both statins and miR-206 treated mice maintained on the normal chow (supplemental Fig. S2A). Consistent with the observation in mice maintained on the HFHC diet, miR-206 significantly reduced hepatic triglycerides, while no significant change in hepatic triglyceride was observed in statins-treated mice (supplemental Fig. S2B). While statins-treated mice showed a slight increase in hepatic cholesterol, miR-206-treated mice exhibited a reduction in hepatic cholesterol levels (supplemental Fig. S2C). Levels of serum cholesterol and triglyceride were reduced in both miR-206- and statins-treated mice (supplemental Fig. S2D, E). MiR-206 also significantly reduced blood glucose, while statins failed to affect this parameter (supplemental Fig. S2F). Regarding lipoproteins, both miR-206- and statins-treated mice maintained on the normal chow diet exhibited reduced VLDL-C and LDL-C levels, while HDL-C levels remained unchanged (supplemental Fig. S2G–I).

To elucidate the miR-206-mediated decline in VLDL-C and LDL-C, mice maintained on a standard diet were administered either AAV8-SCR (control) or AAV8-miR-206. Four weeks post-injection, mice underwent a five-hour fast, and hepatic VLDL secretion rate was assessed by administering Triton WR1339 to inhibit lipolysis and ^35^S methionine/cysteine to label proteins. One hour after Triton WR1339 injection, VLDL-triglyceride production rate markedly decreased in miR-206-treated mice (supplemental Fig. S2J). FPLC analysis unveiled that the reduction in serum triglycerides post-Triton WR1339 was solely observed in the VLDL fraction (supplemental Fig. S2K). In summary, both miR-206 and statins reduce serum triglycerides and cholesterol, as well as VLDL-C and LDL-C levels. MiR-206 also lowers hepatic triglycerides, hepatic cholesterol, and blood glucose, whereas statins increase hepatic cholesterol and exhibit no effect on blood glucose.

### Statins promoted fecal neutral sterol excretion while miR-206 slightly reduced this process

**Fig. 7:**
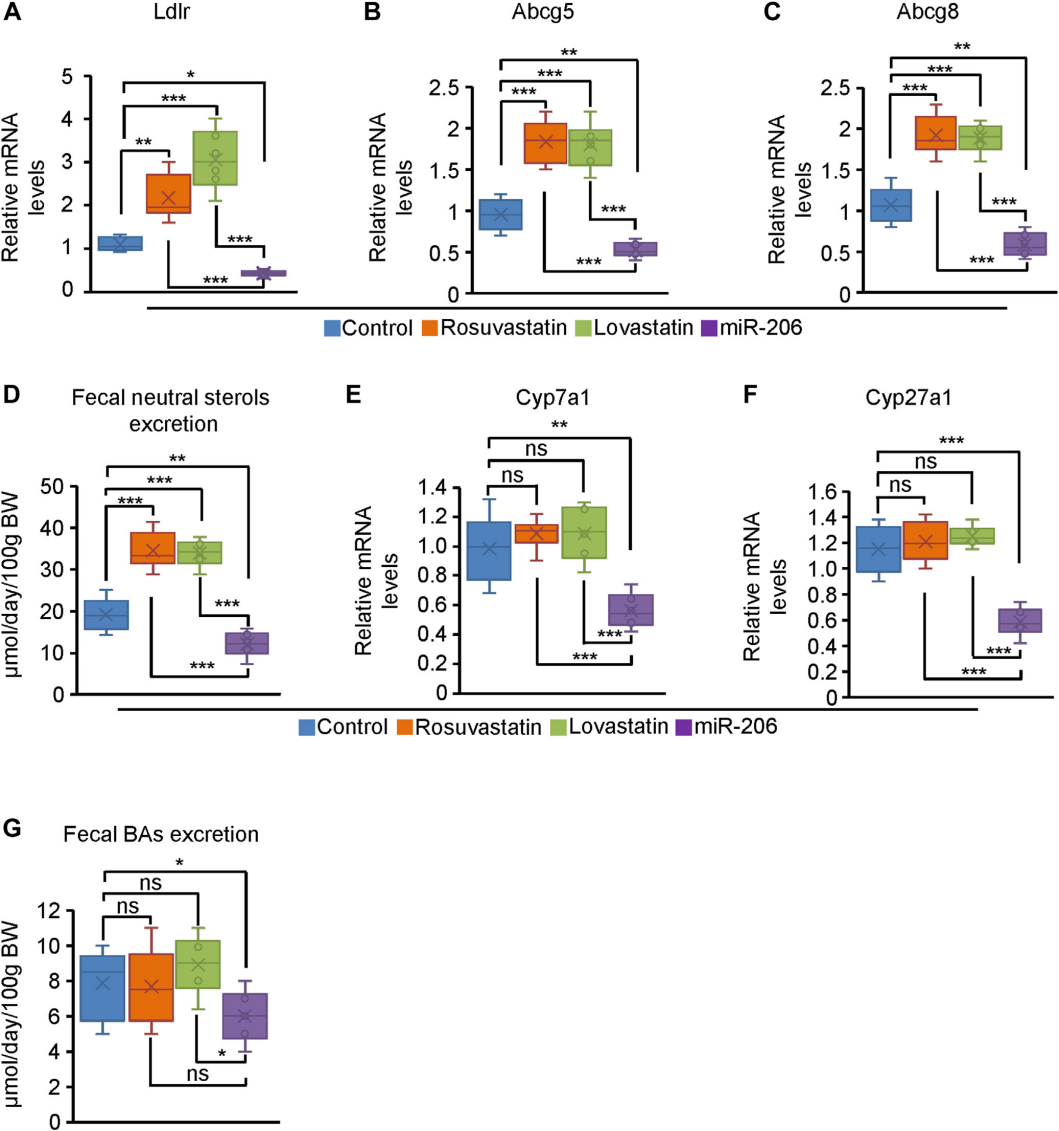
The rates of fecal BAs and neutral sterols excretion in the HFHC-treated mice subjected to statin or miR-206 treatment. Eight-week-old wild-type male C57BL/6J mice were kept on the HFHC diet for eight weeks. At 16 weeks of age, mice were treated with MC-SCR (control, n = 6), rosuvastatin (n = 6), lovastatin (n = 6), and MC-miR-206 (n = 6) for eight weeks. Livers and feces were collected for the following analysis. A–C: mRNA levels of *Ldlr*, *Abcg5*, and *Abcg8* in hepatocytes isolated from four groups of mice treated with MC-SCR, rosuvastatin, lovastatin, and MC-miR-206. D: Levels of fecal neutral sterols excretion in four groups of mice. E, F: mRNA levels of *Cyp7a1* and *Cyp27a1* in hepatocytes of four groups of mice. G: Levels of fecal BAs excretion in four groups of mice. Data represent mean ± SD. ∗*P* < 0.05, ∗∗*P* < 0.01, ∗∗∗*P* < 0.001 and ns: no significance (A–G: one-way ANOVA test).

Elimination of cholesterol from the body also takes place through two major pathways. ∼ 40% of total cholesterol removal occurs by its conversion to bile acids (BAs), a process that takes place exclusively in the liver. (44) During this process, water-insoluble cholesterol is converted into water-soluble BAs. The second pathway of cholesterol removal is the secretion of cholesterol by the liver into the bile, a process coupled with the secretion of BAs. (44) The LDL receptor (LDLR) is a transporter that mediates hepatic uptake of cholesterol and the heterodimer of ABCG5 and ABCG8 controls cholesterol export. (44) Statins promoted expression of *Ldlr*, *Abcg5* and *Abcg8* (Fig. 7A–C). Fecal neutral sterol excretion was significantly increased in statins-treated mice (Fig. 7D). In contrast, levels of *Ldlr*, *Abcg5* and *Abcg8* and fecal neutral sterol excretion were reduced in miR-206-treated mice (Fig. 7A–D). It is known that cholesterol increases expression of *Abcg5* and *Abcg8*, (45) while miR-206 significantly reduced hepatic cholesterol by targeting *Hmgcr* (Fig. 5C). It is our speculation that the reduction in hepatic cholesterol due to miR-206 treatment led to reduced mRNA levels of *Abcg5* and *Abcg8* and fecal neutral sterol excretion in miR-206-treated mice (Fig. 7A–D). Given the important role of LDLR in the hepatic uptake of cholesterol, we further analyzed the effects of miR-206 on LDLR protein and cholesterol uptake in hepatocytes. Levels of LDLR protein and cholesterol uptake were significantly increased in hepatocytes isolated from statins-treated mice, while these parameters were slightly reduced in hepatocytes of miR-206-treated mice (supplemental Fig. S3A–C). To explain this observation, we further analyzed target genes of miR-206. Unexpectedly, we observed that *Srebf2* which encodes SREBP2, a transcription activator of *Ldlr*, is a potential target of miR-206 (46). It is our speculation that miR-206 reduced *Ldlr* by directly targeting *Srebf2* in miR-206-treated mice.

BAs synthesis generates bile flow which is important for biliary secretion of cholesterol. (47) The synthesis of BAs has traditionally been shown to occur via two pathways. Cholesterol 7α-hydroxylase (CYP7A1) performs the initial and rate-limiting step in the classical pathway and sterol 27-hydroxylase (CYP27A1) initiates the hydroxylation of cholesterol in the alternative pathway. (48) Statins failed to alter the expression of *Cyp7a1* and *Cyp27a1* and fecal BAs excretion (Fig. 7E–G). In contrast, expression of *Cyp27a1* and *Cyp7a1* and fecal BAs excretion were reduced in miR-206-treated mice (Fig. 7E–G). Cholesterol induces the expression of *Cyp7a1* and *Cyp27a1*. (49, 50) As described above, miR-206 significantly reduced hepatic cholesterol, which potentially explained reduced mRNA levels of *Cyp7a1* and *Cyp27a1* and fecal BAs excretion in miR-206-treated mice. Similarly, in mice maintained on the normal chow, statins increased expression of *Hmgcr*, *Ldlr*, *Abcg5*, and *Abcg8* and fecal neutral sterols excretion, while miR-206 reduced these parameters (supplemental Fig. S4A–E). Statins failed to significantly change the expression of *Cyp7a1* and *Cyp27a1* and fecal BAs excretion, while miR-206 reduced these parameters (supplemental Fig. S4F–H). In sum, statins promote fecal neutral sterols excretion but do not affect fecal BAs excretion, while these parameters are reduced in miR-206-treated mice.

## Discussion

Lowering circulating lipids and cholesterol through dietary or pharmacological therapy is mandatory to prevent premature CVD. Statins are the first-line therapy for the treatment of hypercholesterolemia. MAFLD, as another metabolic disorder, is prevalent among patients with hyperlipidemia. (51) Hypertriglyceridemia, MAFLD, and hypercholesterolemia are highly interconnected metabolic disorders. (51) Up to date, no FDA-approved drugs are available for the treatment of MAFLD. In some hyperlipidemia individuals, statins alone or in combination fail to achieve therapeutic goals, especially for the prevention of CVD, highlighting the needs for developing new cholesterol-lowering agents. Statins and other lipid-lowering drugs for the treatment of hyperlipidemia function mainly by promoting the removal of circulating cholesterol and lipid. (52) Considering that hepatic DNL and cholesterol synthesis are the major sources of hepatic and circulating lipid and cholesterol, (53) it is important to develop a therapeutic drug that can simultaneously target hepatic DNL and cholesterol synthesis for the treatment of hypertriglyceridemia and hypercholesterolemia. In this study, we showed that miR-206 is able to drive a significant reduction of hypercholesterolemia, hypertriglyceridemia, hepatosteatosis, and hyperglycemia by inhibiting cholesterol synthesis, hepatic DNL and gluconeogenesis, in addition to alleviating hepatic toxicity, DNA damage and ER and oxidative stress. This study provides several unique characteristics of miR-206 as a potential medication.

First, we established that MC is an effective approach to delivering miR-206 to the liver, exhibiting low adverse effects and off-target expression. Delivery efficiency affects how a drug exerts its efficacy and even becomes a key factor in determining the success or failure of drug development. The technologies for manufacturing inhibitors and mimics of miRNAs are mature. (13, 14, 15, 16, 54) Considering the potential off-target effects of miRNA mimics and inhibitors in other organs, in this study we used MC to deliver miR-206 to hepatocytes. (55) Hepatocytes, the major site of DNL, cholesterol synthesis, and gluconeogenesis, are terminally differentiated. These long-lived cells with a low turnover rate are well suited for non-integrating MC vectors. In addition, the sizes of both MC and miRNAs are very small, suggesting high delivery efficiency of miR-206 to the liver. We have established that MC-miR-206 was efficiently delivered to hepatocytes of mice and sustained high expression of miR-206 for at least two weeks after one injection of MC-miR-206. Combining MC and *TTR* promoter, miR-206 was highly and specifically expressed in the hepatocytes of mice. We did not detect the expression of miR-206 in other organs after MC-miR-206 injection. Compared to viral vectors, MC does not contain bacterial sequences and transcriptional units that may contribute to an immune response. In addition, it is well-established that MC maintains a circular episome after injection into mice, (56) indicating a low risk of insertional mutagenesis of MC-miR-206. MC is an effective vehicle to specifically deliver miR-206 to the liver.

Second, miR-206 exhibits low hepatic toxicity. As described above, miRNAs are naturally occurring small RNAs to precisely fine-tune multiple pathways, suggesting the potentially low toxicity of miRNAs. As expected, MC-miR-206 treatment significantly reduced hepatic toxicity, DNA damage, and ER and oxidative stress. It is known that hepatic accumulation of cholesterol and triglycerides is the contributor of hepatic injury. MiR-206 significantly reduced hepatic cholesterol and triglycerides, which partially explains the reduction in hepatic toxicity in miR-206-treated mice. In addition, miR-206 significantly reduced levels of MDA and oxo-8-dG and expression of genes controlling MDA production and DNA damage, indicating that miR-206 might represent a unique agent to prevent oxidative stress and DNA damage. Statins have been associated with several side effects including hyperglycemia, elevated hepatic cholesterol and liver injury with autoimmune features. (57) MiR-206 might be superior to statins in the aspect of adverse effects.

Third, miR-206 is a potent inhibitor of hepatic cholesterol synthesis. Overexpression of miR-206 significantly reduced mRNA and protein levels of *HMGCR* and cholesterol synthesis. Two conserved miR-206 binding sites within the 3′UTRs of both human and mouse *HMGCR* were confirmed, (19) providing a mechanistic explanation regarding the potent inhibitory effect of miR-206 on cholesterol synthesis. (19) The liver represents the main site for de novo cholesterol synthesis contributing to approximately 80% of total cholesterol synthesis in mammals. (58) Furthermore, the primary sources of cholesterol in humans are de novo cholesterol synthesis (∼70%) and dietary intake (∼30%). (53) miR-206 is a potent inhibitor of hepatic cholesterol synthesis. In contrast, statins remove cholesterol by driving fecal-neutral steroid excretion. These observations indicate that miR-206 might be more effective than statins in treating hypercholesterolemia patients.

Finally, in addition to alleviating MAFLD, body weight, hypercholesterolemia, and hyperlipidemia, miR-206 also exhibited a strong therapeutic effect on hepatocellular carcinoma (HCC). (20, 21, 22, 23) Cholesterol is a vital component of cellular membranes, making it essential for the growth of cancer cells. (59) Intracellular cholesterol levels appear more important than dietary cholesterol in cancer development. (60) In this study, miR-206 significantly inhibited hepatic cholesterol synthesis and reduced hepatic cholesterol. Consistent with this observation, miR-206 fully prevented HCC development by targeting *Hmgcr*, while all control mice died within 8 weeks after c-Myc injection. (19) These findings provided another layer of evidence that hypercholesterolemia is tightly connected with liver cancer. miR-206 promotes M1 polarization of Kupffer cells (KCs) via targeting *Klf4*, which enhances anti-tumor immunity. (22) M1 polarization of macrophages primarily depends on glycolysis. (61) miR-206, by driving glycolysis, (18) promotes M1 polarization of KCs and thereby exerts anti-tumor immunity. In sum, miR-206 links both cholesterol and glucose metabolism to HCC development.

While miR-206 holds promise as a potential therapeutic agent for treating hypercholesterolemia, it is essential to address several limitations in future studies. The first limitation is that miRNAs can simultaneously modulate multiple genes. Therefore, it is important to analyze the targetome of miR-206 and exclude the effects of miR-206 on other target genes. Our next step will analyze the targetome of miR-206 via HITS-CLIP (high-throughput sequencing of RNA isolated by crosslinking immunoprecipitation). Another pitfall is the off-target effects of miR-206. Notably, within MAFLD and/or hyperlipidemia patients, the upregulation of HMGCR and LXRα specifically in hepatocytes, as opposed to other organs, is a remarkable observation. This organ-specific pattern simplifies the task of circumventing miRNAs' inherent limitations in governing diverse gene targets, achieved by optimizing the dosage of miRNAs. In addition, we have combined mini-circle and *TTR* promoter to ensure liver-specific expression of miR-206.

In sum, MC-miR-206 exhibited a potent therapeutic effect on hepatosteatosis, hyperglycemia, hypertriglyceridemia, and hypercholesterolemia by inhibiting DNL, gluconeogenesis, and cholesterol synthesis. In contrast, statins exhibited therapeutic effects on hypercholesterolemia by driving fecal-neutral steroid excretion in mice. Notably, MC-miR-206 significantly alleviated hepatic toxicity, DNA damage, and oxidative stress.

Supplementary methods and data are available for this manuscript. (62, 63, 64, 65).

## Data availability

All the data have been included in the manuscript.

## Supplemental data

This article contains supplemental data (62, 63, 64, 65, 66).

## Conflict of interests

The authors involved in this study declared that they have nothing to disclose regarding funding or conflict of interest with respect to this manuscript.
