# Supplementary material for: MicroRNA-206 as a potential cholesterol-lowering drug is superior to statins in mice
Source: J Lipid Res. 2024 Jun 10;65(7):100576. doi: 10.1016/j.jlr.2024.100576 (PMC11292365; doi:10.1016/j.jlr.2024.100576)
Supplement: Supporting information [file mmc1.docx]

**Supplementary Materials and Methods**

**HMGCR enzyme activity assay**

HMGCR enzyme activity in hepatocytes were measured using HMG-CoA Reductase Activity Assay Kit (Colorimetric) (ab204701) according to the manufacturer’s protocols. Hepatocytes were isolated in our previous publication ([1](#_ENREF_1)).

**8-oxo-dG (8-oxo-2'-deoxyguanosine) analysis in the liver**

Levels of 8-oxo-dG analysis in the liver were analyzed as described previously ([2](#_ENREF_2)). Specially, nuclear DNA from the liver was isolated via DNA Extractor WB kit (Wako Chemicals). 100 mg of liver were homogenized in a Dounce homogenizer in ice-cold lysis solution. Nuclei were collected by centrifuging the homogenate at 10,000 g for 20 s, and the nuclear pellets were re-suspended in the enzyme reaction solution and proteinase K (10 µg/ml) provided with the kit. RNase mixture (Ambion, Austin, TX) was then added to a final concentration of 20 µg/ml. In total, 50–75 µg of nuclear DNA were hydrolyzed. The 8-oxo-2-deoxyguanosine (oxo8dG) and 2-deoxyguanosine (2-dG) were resolved by high pressure liquid chromatography and quantified by electrochemical detection. The data are expressed as the ratio of nmol of 8-oxo-dG to 10^5^ nmol of 2-deoxyguanosine (2dG).

**MDA analysis in the liver**

Levels of MDA in the liver were measured using Lipid Peroxidation (MDA) Assay Kit (Colorimetric) (AbCam, ab233471) according to the manufacturer’s protocols.

**RNA isolation and quantitative reverse transcription-PCR (qRT-PCR)**

Total RNA was isolated with miRNeasy Mini Kit (Qiagen). To assess gene expression, 1 μg RNA was used for cDNA synthesis with Superscript III reverse transcription reagent (Invitrogen). PCR amplification was performed at 50°C for 2 minutes and 95°C for 10 minutes, followed by 40 cycles at 95°C for 15 seconds and 60°C for 1 minute in a 7900 real time-PCR system with SYBR green (Applied Biosystems). For each sample, we analyzed β-actin, GAPDH or 18S rRNA expression to normalize target gene expression. Primers for qRT-PCR were designed with Primer Express software (Applied Biosystems).

To determine levels of miRNA expression, 10 ng RNA were used for miRNA-specific cDNA synthesis with the TaqMan MicroRNA Reverse Transcription Kit and Taqman MicroRNA Assays (all Applied Biosystems). PCR amplification was performed at 95°C for 10 minutes, followed by 40 cycles at 95°C for 15 seconds and 60°C for 1 minute in a 7900 real time-PCR system (Applied Biosystems). The small RNA Sno202 and RNU6 were used to normalize target miRNA expression. Relative changes in gene and miRNA expression were determined using the 2^-ΔΔCt^ method ([3](#_ENREF_3)).

**Hepatic lipid analysis**

Mouse liver (100 mg) was placed in 1 mL chloroform/methanol (2:1) mixture and incubated on mice for 10 minutes before homogenization. Lipids were extracted from liver homogenates through room temperature orbital shaking (2 hours) followed by centrifugation (5000 RPM for 5 minutes). Supernatants were collected and washed with 0.4 mL chloroform/methanol (2:1) mixture by centrifugation at 5000 RPM for 20 minutes (room temperature). New supernatants were washed with 0.2 volume of 0.9% NaCl. After centrifuging for 5 minutes at 5000 RPM, supernatants were removed and lower-phase was dried at 42°C. Dried lipids were re-suspended in 2% Triton X-100. Liver triglycerides were quantified via a colorimetric assay using a triglyceride assay kit from Roche Diagnostics according to the manufacturer’s protocols.

**Analysis of fecal bile acids excretion**

Feces from mice were collected during a 72 hours period prior to termination. 5 mg of dried feces was used for extraction of bile acids as described previously.([4](#_ENREF_4)) To extract neutral sterols from feces, 10 mg of dried feces (10 mg) were treated with 1 mL alkaline methanol using 5α-cholestane as internal standard. After treatment, the neutral sterols were extracted three times with 3 mL of petroleum ether.([4](#_ENREF_4)) Quantification of bile acid and neutral sterols were performed as described previously.([4](#_ENREF_4))

**Blood lipid analysis**

Blood was collected into tubes from cardiac puncture of mice. Serum was separated by centrifugation (3000 x RPM for 20 min at 4 °C and triglyceride (mg/dL, Roche Diagnostics) was quantified enzymatically. Serum chemistry was carried out by the Pathology Laboratory of the University of Minnesota or Shanxi Medical University.

**Analysis of HDL-C, LDL-C and VLDL-C**

Serum was collected by centrifugation of blood at 8,000g using microtainer serum separator tubes (BD Biosciences, San Jose, CA) for 15 min at 4 °C and stored at -80 °C for further analysis. Levels of serum VLDL-C, LDL-C and HDL-C were analyzed using Mouse VLDL ELISA Kit (MyBioSource, MBS3807366), mouse LDL ELISA Kit (CrystalChem, Cat. No: 79980) and mouse HDL-C ELISA Kit (MyBioSource, Cat.No. 79990) according to the manufacturer’s instructions.

**Triton WR1339 treatment of mice**

Eight-week old male C57BL/6J mice kept on the standard diet were then injected with MC-SCR (*n*=6) or MC-miR-206 (*n*=6). Four weeks after injection, mice were fasted for 5 hours to minimize the contribution of postprandial lipid to the plasma triglycerides concentrations. After fasting, mice were injected intraperitoneally with Triton WR1339 (500 mg/kg) and 500 µCi of ^35^S-methionine/cysteine in saline to inhibit lipolysis and to label newly synthesized proteins. VLDL clearance is completely inhibited under these conditions,([5](#_ENREF_5)) allowing us to determine hepatic production rate of VLDL. Therefore, blood was taken before injection (50 μL) and at 1 hour after Triton WR1339 injection. Serum triglycerides levels were measured using an enzyme method (Wako, Osta, Japan). The VLDL-triglycerides production rate was calculated by the increase in serum triglycerides level from baseline to 1 hour after WR1339 injection. We used a 1-hour time point to estimate the rate of VLDL-triglycerides production, assuming a linear increase of serum triglycerides concentration during this period. The data were expressed as micromoles of triglycerides produced per hour per kilogram of body weight, assuming a serum volume of 3.5% (liters per kilogram). VLDL was isolated by ultracentrifugation, run on an SDS-PAGE gel and ApoB100 bands cut and counted for quantitation of VLDL secretion.

**FPLC analysis**

Blood of mice was collected for serum isolation. After serum isolation by centrifugation, the distribution of lipids within the serum lipoprotein fractions was assessed by FPLC. Pooled serum from mice was used for FPLC analysis.

**Histological analysis**

Liver samples were embedded in Tissue-Tek OCT embedding compound, and frozen on dry ice. 8 µm-thick sections were cut with a Leica CM3050 S cryostat, air-dried, and fixed in 10% formalin. After washing, sections were stained with an Oil-Red-O (Sigma-Aldrich)/60% isopropanol solution (Fisher Scientific). Briefly, sections were rinsed with 60% isopropanol and stained for 20 min with prepared Oil Red O solution (0.5% in isopropanol followed by dilution to 60% with distilled water and filtered).

**Western blot analysis**

Western blot was performed following standard procedures and analyzed by LICOR-Odyssey infra-red scanner. Primary antibodies of HMGCR (ThermoFisher Scientific, Cat. No.: MA5-35242), G6PC (Novus Biologicals, NBP1-80533), LDLR (R&D Systems, Inc., AF2255-SP), Srebp1c (Abcam, Cat. No.: ab313881), Srebp1c (Invitrogen, Cat. No.: PA5-120378) and SREBP2 (Abcam, Cat. No.: ab194667) were purchased.

**
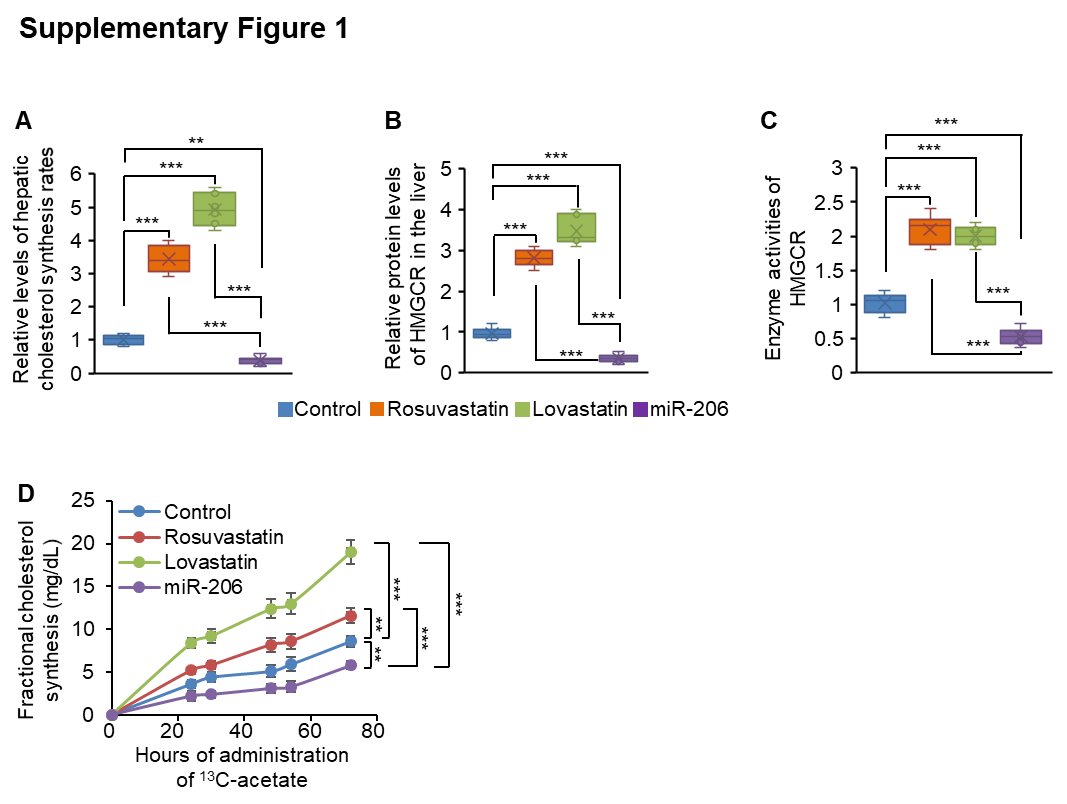
Supplementary Figures**

**Supplementary Figure 1 miR-206 inhibited but statins promoted hepatic cholesterol synthesis in mice kept on the normal chow.** (**A**) Relative levels of hepatic cholesterol synthesis rates in four groups of mice, as revealed by deuterium oxide assay. Eight-week-old wild-type male C57BL/6J mice kept on the normal chow were treated with statins or MC-miR-206 for two weeks. Mice were then injected with 99% deuterium oxide. 60 min after injection, mice were terminated by cardiac puncture. (**B**) Levels of HMGCR protein in four groups of mice as revealed by ELISA. (**C**) Levels of HMGCR enzyme activities in livers of four groups of mice. **(D)** Total body cholesterol synthesis measured by the incorporation of ^13^C-acetate into the cholesterol molecule over a period of 72 hours in mice treated with MC-SCR (control, *n*=6), rosuvastatin (*n*=6), lovastatin (*n*=6) or MC-miR-206 (*n*=6). Eight-week-old wild-type male C57BL/6J mice kept on the normal chow were treated with statins or MC-miR-206 for two weeks. After two weeks of statin or MC-miR-206 treatment, 2% ^13^C-acetate was added to the drinking water ad libitum for 72 h. Bloodspots were taken at time points 0, 24, 31, 48, 55, and 72 h. Data represent mean ± SD. ***p* < 0.01 and ****p* < 0.001 (Figure 1A-D: one-way ANOVA test).

**
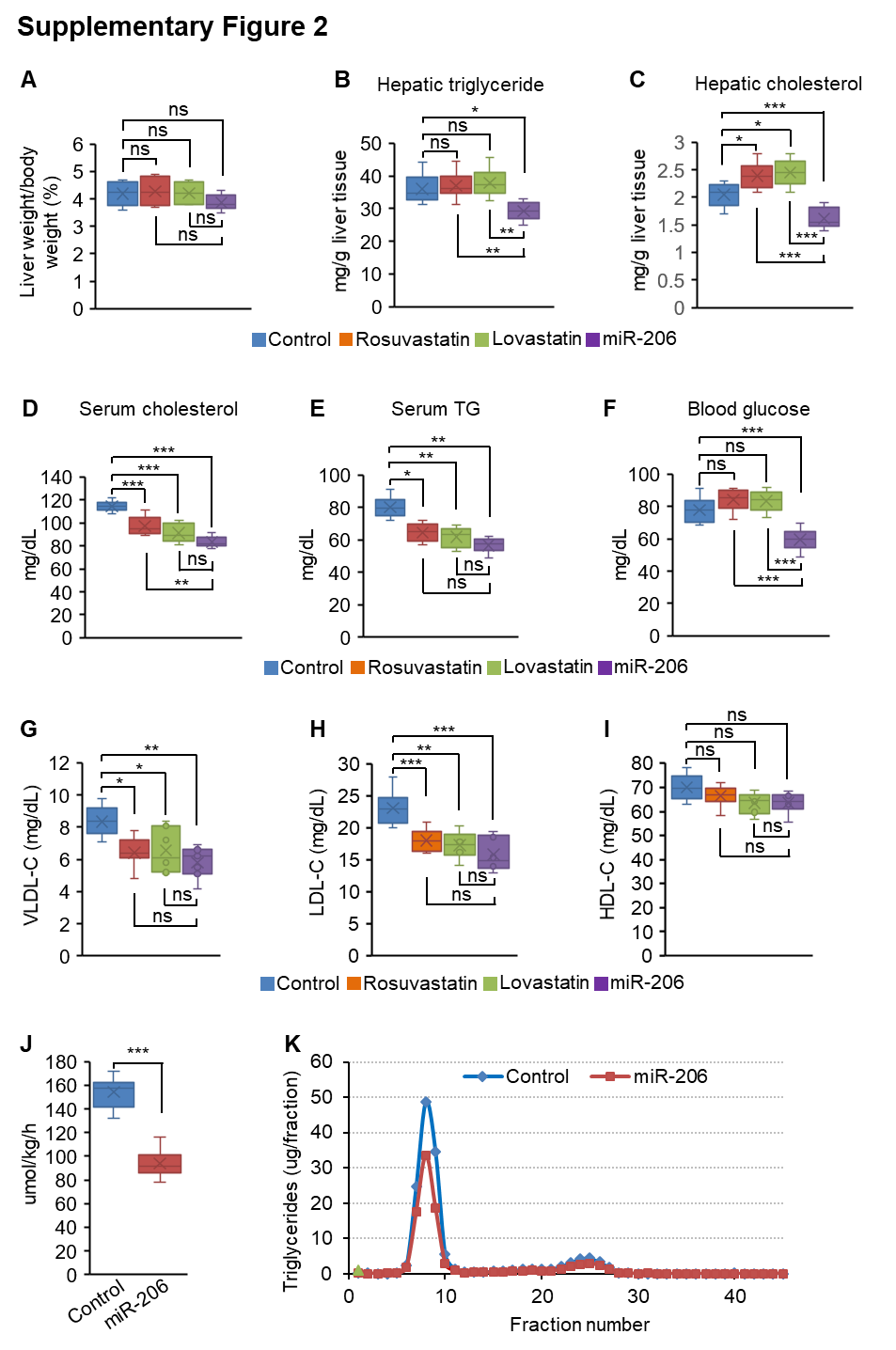
**

**Supplementary Figure 2 The effects of MC-miR-206 and statins on hepatic and serum triglyceride and cholesterol as well as blood glucose in mice kept on the normal chow.** Eight-week-old C57BL6/J mice kept on the normal chow were treated with MC-SCR (*n*=6), MC-miR-206 (*n*=6), rosuvastatin (*n*=6), or lovastatin (*n*=6) for eight weeks. After that, livers and blood were collected for analysis. (**A**) Ratios of the liver weight to body weight; (**B-C**) Levels of hepatic TG and cholesterol; (**D-E**) Levels of total serum triglycerides (TG) and cholesterol; (**F**) Level of blood glucose; and (**G-I**) Levels of HDL cholesterol (HDL-C), VLDL cholesterol (VLDL-C) and LDL cholesterol (LDL-C). (**J**) The rates of VLDL production in mice treated with MC-SCR or MC-miR-206. The difference between baseline and 1-hour serum triglyceride levels was measured after Triton WR1339 treatment. (**K**) Levels of VLDL-triglyceride in pooled serum from mice treated with MC-SCR or MC-miR-206, as revealed by FPLC. Data represent mean ± SD. **p* < 0.05, ***p* < 0.01, ****p* < 0.001 and ns: no significance (Figure 2A-I: one-way ANOVA test).

**
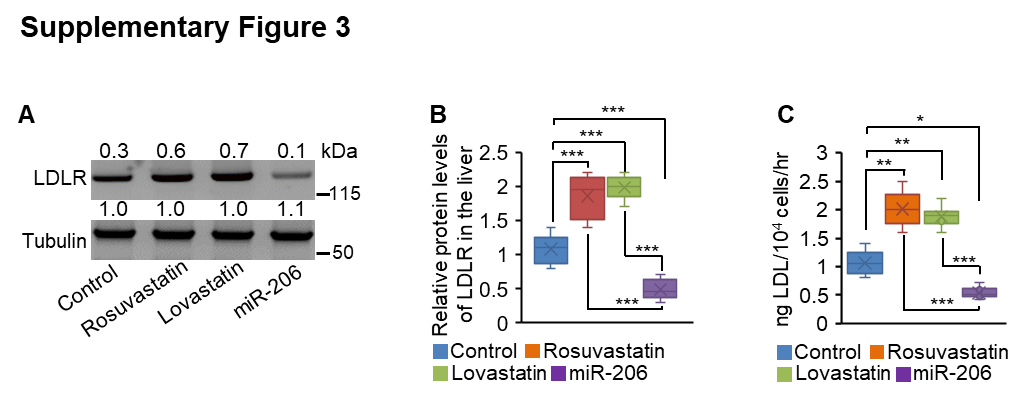
**

**Supplementary Figure 3 Statins promoted LDLR expression and cholesterol uptake in hepatocytes, while miR-206 exhibited the opposite effects.** Eight-week-old wild-type male C57BL/6J mice were kept on the HFHC diet for eight weeks. At 16 weeks of age, mice were treated with MC-SCR (control, *n*=6), rosuvastatin (*n*=6), lovastatin (*n*=6), and MC-miR-206 (*n*=6) for eight weeks. Hepatocytes were isolated by perfusion. **(A**) Protein levels of LDLR in pooled hepatocytes as revealed by Western blot; **(B)** Protein levels of LDLR in hepatocytes isolated from four groups of mice, as revealed by ELISA; and (**C**) Labeled LDL uptake in hepatocytes isolated from four groups of mice. Data represent mean ± SD. **p* < 0.05, ***p* < 0.01, ****p* < 0.001 (Figure 3A-C: one-way ANOVA test).

**
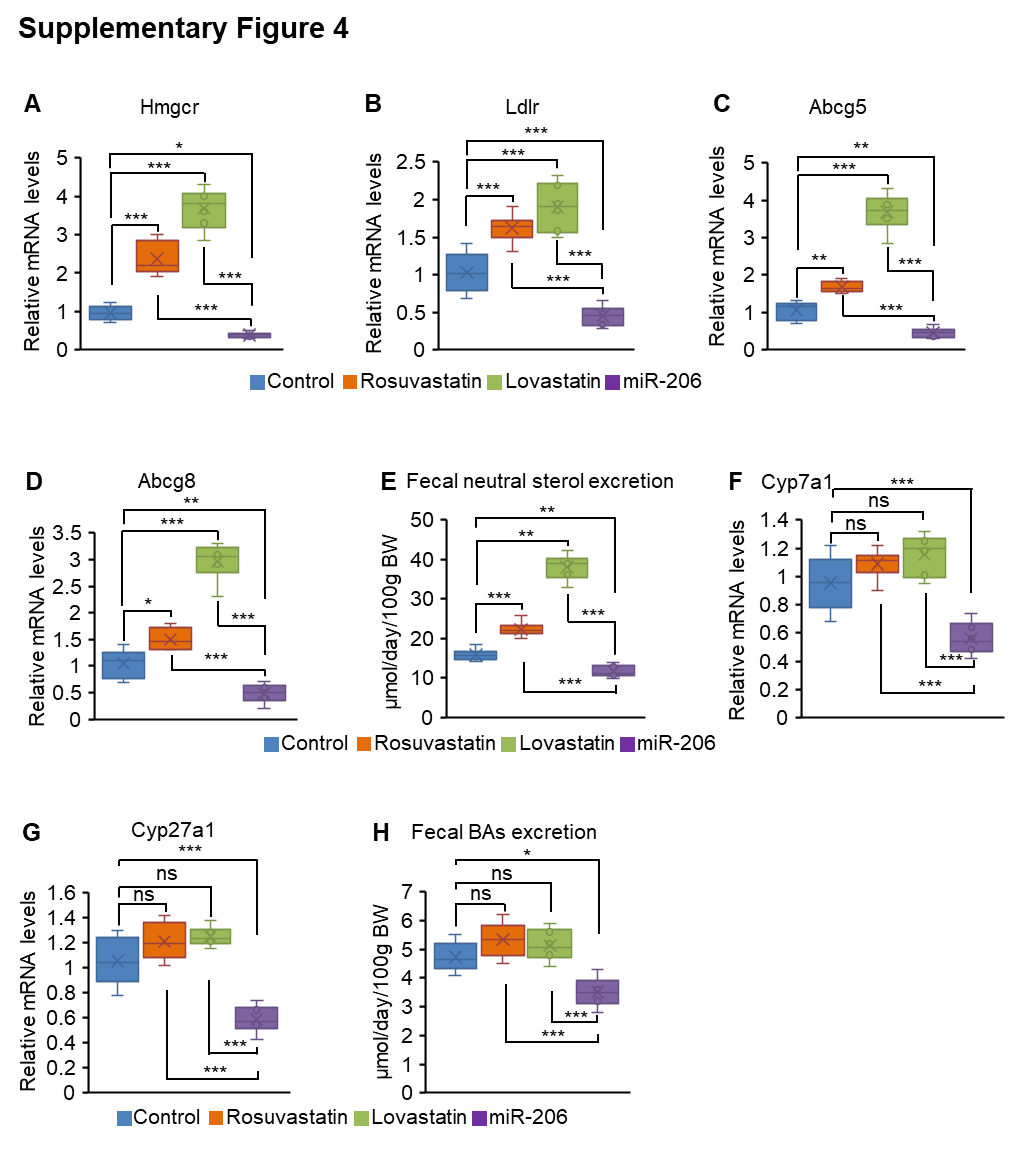
**

**Supplementary Figure 4 The effects of miR-206 and statins on levels of fecal BAs and neutral sterol excretion in mice kept on the normal chow.** Eight-week-old wild-type male C57BL/6J mice kept on the normal diet were treated with MC-SCR (control, *n*=6), rosuvastatin (*n*=6), lovastatin (*n*=6), and MC-miR-206 (*n*=6) for eight weeks. (**A-D**) mRNA levels of *Hmgcr*, *Ldlr*, *Abcg5* and *Abcg8* in livers of four groups of mice treated with MC-SCR (control, *n*=6), rosuvastatin (*n*=6), lovastatin (*n*=6), and MC-miR-206 (*n*=6). (**E**) Levels of fecal neutral sterol excretion in four groups of mice. (**F-G**) mRNA levels of *Cyp7a1* and *Cyp27a1* in four groups of mice. (**H**) Levels of hepatic BAs excretion in four groups of mice. Data represent mean ± SD. ***p* < 0.01, ****p* < 0.001 and ns: no significance (Figure 5A-H: one-way ANOVA test).

**References**

1. Zhang T, Duan J, Zhang L, Li Z, Steer CJ, Yan G, Song G. LXRα promotes hepatosteatosis in part through activation of microRNA‐378 transcription and inhibition of Ppargc1β expression. Hepatology. 2019;69(4):1488-503.

2. Hamilton ML, Van Remmen H, Drake JA, Yang H, Guo ZM, Kewitt K, Walter CA, Richardson A. Does oxidative damage to DNA increase with age? Proceedings of the National Academy of Sciences. 2001;98(18):10469-74.

3. Schmittgen TD, Livak KJ. Analyzing real-time PCR data by the comparative C(T) method. Nature Protocol. 2008;3(6):1101-8. PubMed PMID: 18546601.

4. Post SM, de Crom R, van Haperen R, van Tol A, Princen HM. Increased fecal bile acid excretion in transgenic mice with elevated expression of human phospholipid transfer protein. Arteriosclerosis, Thrombosis, and Vascular Biology. 2003;23(5):892-7.

5. Aalto-Setälä K, Fisher E, Chen X, Chajek-Shaul T, Hayek T, Zechner R, Walsh A, Ramakrishnan R, Ginsberg H, Breslow J. Mechanism of hypertriglyceridemia in human apolipoprotein (apo) CIII transgenic mice. Diminished very low density lipoprotein fractional catabolic rate associated with increased apo CIII and reduced apo E on the particles. J Clin Invest. 1992;90(5):1889-900.
